# Blister formation in acute compartment syndrome: Unraveling the underlying predictors

**DOI:** 10.1097/MD.0000000000038191

**Published:** 2024-05-17

**Authors:** Yubin Long, Shuo Yang, Junfei Guo, Zhiyong Hou

**Affiliations:** a Department of Orthopaedic Surgery, The Third Hospital of Hebei Medical University, Shijiazhuang, Hebei, P.R. China; b Orthopaedic Research Institute of Hebei Province, Shijiazhuang, Hebei, P.R. China; c The Third Department of Orthopedics, Baoding First Central Hospital, Hebei, P.R. China; d Department of Joint Surgery, Honghui Hospital, Xi’an Jiaotong University, Xi’an, China; e NHC Key Laboratory of Intelligent Orthopaedic Equipment (The Third Hospital of Hebei Medical University), Shijiazhuang, Hebei, P.R. China.

**Keywords:** acute compartment syndrome, alcohol, blisters, PLR, referral, seasonal factors

## Abstract

Blisters are a common complication of orthopedic trauma and can cause surgery delay and increase the risk of infection. This study aims to identify risk factors for blisters in patients with acute compartment syndrome (ACS). Our study collected data from 206 ACS patients admitted to 2 hospitals between November 2013 and January 2021. Patients were divided into 2 groups: the blister group (BG) and the control group (CG), based on the presence or absence of blisters. We conducted univariate analysis, logistic regression analysis, and receiver operating characteristic (ROC) curve analysis to identify any significant differences in demographics, comorbidities, and admission laboratory test results between the 2 groups. Our study found that the incidence of blisters in ACS patients was 21.8% (45 out of 206). Univariate analysis identified several factors that were significantly associated with blister formation. Logistic regression analysis showed that patients who developed ACS in the winter or spring (*P* = .007, OR = 2.690, 95% CI [1.308–5.534]), patients who received a referral (the process whereby patients are transferred between medical facilities for further evaluation and treatment attempts prior to admission to our hospital) (*P* = .009, OR = 4.235, 95% CI [1.432–12.527]), and patients with higher PLR (*P* = .036, OR = 1.005, 95% CI [1.000–1.009]) were independent risk factors for blisters. Additionally, a history of drinking (*P* = .039, OR = 0.027, 95% CI [0.046–0.927]) was found to be a protective factor for blister formation in these patients. Moreover, ROC curve analysis showed that a PLR value of 138 was the cutoff point for predicting the development of blisters in ACS patients. Our study identified seasonal factors (refer to these months like winter or spring), referral, and patients with higher PLR as independent risk factors, and a history of drinking as a protective factor for blister formation in ACS patients. These findings allow clinicians to individualize the evaluation of blister risk and perform early targeted therapies.

## 1. Introduction

Acute Compartment Syndrome (ACS) emerges as a critical condition following fractures or other traumas within an enclosed fascial space.^[1,2]^ Although its occurrence is relatively infrequent, with an annual incidence rate of 3.1 per 100,000 individuals, ACS predominantly affects males over females at a ratio of 10:1.^[3,4]^ The repercussions of belated or inappropriate intervention are dire, potentially leading to functional impairment or, in extreme cases, mortality.^[5]^ At the heart of ACS lies a pathophysiological cascade initiated by reperfusion-induced swelling post-trauma, escalating to increased pressure within the compartment and consequent tissue death.^[6]^ Predominantly targeting the legs and forearms, ACS frequently manifests with skin blisters in these areas.^[3,4]^

Blisters represent a prevalent skin condition among hospitalized individuals, occurring in roughly 2.9% of cases. Their emergence can be attributed to a variety of factors, including burns, cupping therapies, and notably, fractures.^[7–11]^ Particularly prone are those regions of the body where the skin is tightly bound to the underlying bone with minimal subcutaneous fat for cushioning, such as the ankles, wrists, elbows, and feet. These areas are frequently subjected to high-energy orthopedic trauma, making them common sites for blister development.^[12]^ The clinical management of blisters following fractures presents several challenges, not least of which include the potential for delayed surgical interventions and a heightened risk of infection.^[13]^ Research has elucidated the pathogenic process of blister formation: during the inflammatory response, swollen soft tissues cause an increase in interstitial pressure. This elevation disrupts the cohesion of epidermal cells, thereby promoting the movement of fluid into the blister. Concurrently, a rise in colloid pressure acts to drive fluid into spaces within or just beneath the epidermis, further exacerbating blister formation.^[14]^

Despite the notable frequency of blister occurrence in hospitalized individuals, the literature remains scant on studies specifically addressing the precursors to blister development in patients with ACS. Thus, our investigation seeks to fill this gap by exhaustively examining the variables linked to the prevalence of blisters among ACS sufferers. Through a retrospective analysis of 206 cases of ACS, our research delineates the significant risk factors contributing to the formation of blisters.

## 2. Materials and methods

### 2.1. Ethics statement

Our investigation encompassed patients diagnosed with ACS who received treatment at the Third Hospital of Hebei Medical University and Baoding No. 1 Central Hospital over the period from November 2013 to January 2021. Ethical clearance was duly obtained from the institutional review boards of both hospitals, aligning with the ethical guidelines stipulated by the 1964 Helsinki Declaration. Furthermore, our study was officially registered with the identifier NCT04529330 and received ethical approval under the reference number S2020-022-1 (2022116).

### 2.2. Patients

**Figure 1.**
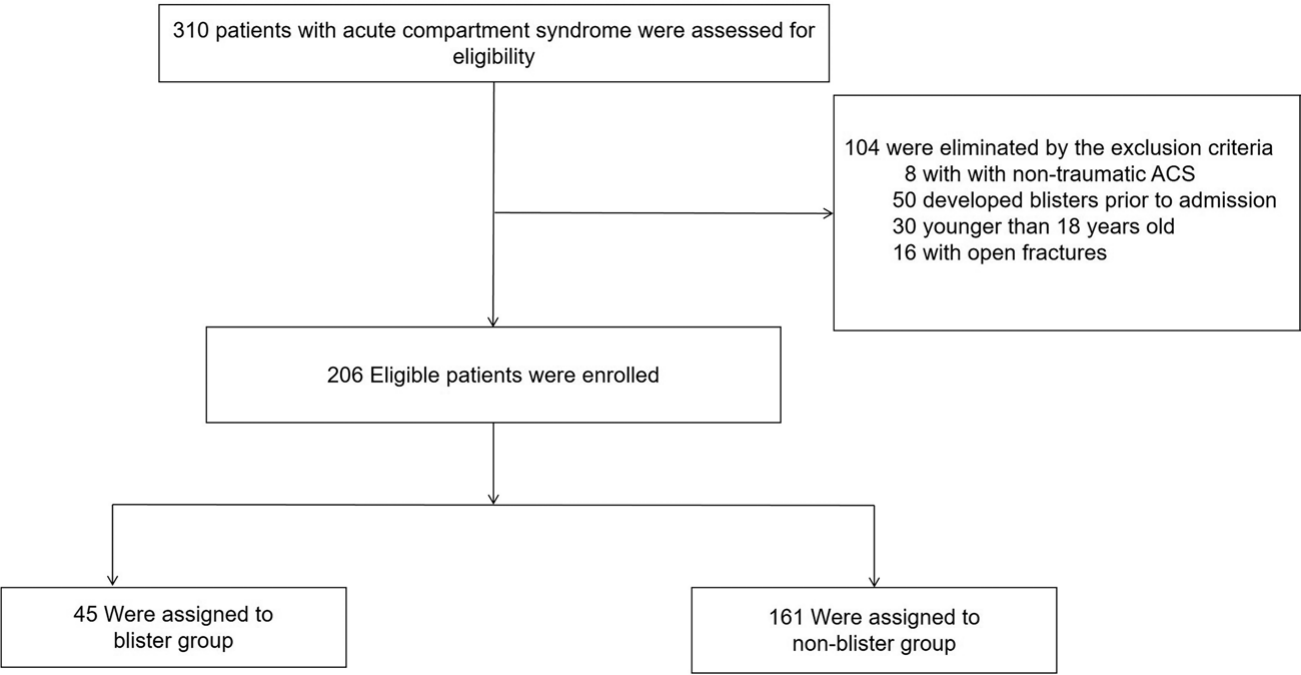
Flow diagram of included patients.

This study was executed at the Third Hospital of Hebei Medical University and Baoding No. 1 Central Hospital. Both institutions are distinguished tertiary hospitals equipped with Level I trauma centers. Data on comorbidities were meticulously extracted from the hospitals’ Electronic Medical Records (EMR) system, following an in-depth examination of patient files. Our analysis focused on patients diagnosed with traumatic ACS who had comprehensive medical records. We strategically excluded individuals presenting with nontraumatic ACS, those who had developed blisters before hospital admission, were under the age of 18, or had sustained open fractures (refer to Fig. 1 for exclusion flowchart). The cohort for this study consisted of 206 patients - 180 men and 26 women - who satisfied these inclusion and exclusion criteria. For the purpose of this research, we categorized these patients into 2 distinct groups: those with blisters (Blister Group - BG) and those without (Control Group - CG).

To circumvent the risk of circular definitions, we established stringent diagnostic criteria for ACS, incorporating classic clinical indicators such as disproportionate pain and paresthesia, augmented by objective measurements of compartment pressure. In instances of subtle symptomatology, direct measurement of muscle tissue pressure was employed. Affected patients were subjected to continuous clinical assessment and hourly monitoring of Intracompartmental Pressure (ICP) over a period of 24 to 48 hours utilizing a continuous ICP slit catheter. The nursing team conducted hourly measurements, with a sustained differential pressure (ΔP) - defined as diastolic blood pressure minus compartment pressure - falling below 30 mm Hg for a duration of 2 hours earmarked as indicative of ACS. This approach ensures the generation of objective and repeatable data, significantly diminishing the influence of subjective interpretation and diagnostic inaccuracies.

Following the confirmation of an ACS diagnosis, the imperative progression to a fasciotomy procedure was highlighted. This surgical maneuver is pivotal in alleviating increased compartmental pressure, thus mitigating the risk of severe outcomes, including muscle necrosis, the potential for limb amputation, and, in extreme cases, fatality. The critical interplay between compartmental pressure and ischemic conditions within the fascial space is identified as a fundamental element contributing to blister development in ACS patients.^[15]^

Throughout their admission and subsequent hospitalization, all patients were treated with cryotherapy and advised on the passive elevation of limbs as preventative strategies against further soft tissue damage or the emergence of blisters. The application of ice serves to dampen the inflammatory response, curtail soft tissue swelling by lowering tissue temperature, and consequently, diminish or prevent blister formation.^[15]^ Moreover, limb elevation promotes venous and lymphatic fluid return, further alleviating edema.^[11]^ Despite these preventative measures, our study observed a blister occurrence rate of 21.8%. In cases where blisters formed and resulted in patient discomfort, skilled surgeons would perform incisions at the blister base, allowing for fluid drainage, followed by sterile dressing application. These dressings were routinely changed until reepithelialization of the base was achieved, often extending beyond a week. Within our cohort, 29 patients underwent this procedure for blister fluid extraction, whereas 16 did not.

In our research, we meticulously evaluated a spectrum of variables that could influence the occurrence of blisters in patients afflicted with ACS. This analysis encompassed an array of demographic factors, patient comorbidities, and laboratory findings at the time of admission. Demographic data scrutinized included age, gender, Body Mass Index, mechanism of injury, American Society of Anesthesiologists (ASA) physical status classification score, season during which admission occurred, referral status, smoking habits, alcohol consumption, the interval between the injury and hospital admission, and the application of any dehydrating agents.

For the purpose of our study, ASA scores were dichotomized into 2 categories: the lower risk groups (grades 1–2) and the higher risk groups (grades 3–4). Likewise, the seasonal factors influencing blister formation were categorized, with summer and autumn being grouped together, and winter and spring merged into another category, allowing for a nuanced exploration of how seasonal variations might impact the risk of blister development in ACS scenarios.

Comorbidities assessed in this study included arrhythmia, diabetes, hypertension, coronary heart disease, hypoproteinemia, anemia, and cerebral infarction. At the time of admission, we also examined various laboratory tests, including basophil, eosinophil, hematocrit, hemoglobin, immature, lymphocyte, mean corpuscular hemoglobin concentration, monocyte, mean platelet volume, neutrophil, platelet, red blood cell, White blood cell, activated partial thromboplastin time, fibrinogen, international normalized ratio, prothrombin time, thrombin time, albumin, alkaline phosphatase, aspartate aminotransferase, alanine transaminase, calcium (Ca), potassium (K), sodium (Na), magnesium (Mg), phosphorus (P), chloride (Cl), globulin, cholinesterase, creatine kinase, creatinine, direct bilirubin, glucose, lactate dehydrogenase, osmotic pressure, triglyceride, total cholesterol (TC), total protein, total carbon dioxide (TCO2), ureophil, and uric acid.

Furthermore, we calculated several ratio metrics, including the neutrophil to lymphocyte ratio (NLR), monocyte to lymphocyte ratio (MLR), and platelet to lymphocyte ratio (PLR). NLR and MLR were obtained by dividing the neutrophil/monocyte count by the lymphocyte count, respectively. The PLR was computed by dividing the platelet count by the lymphocyte count. By examining these factors, we aimed to identify independent risk factors for blister formation in ACS patients.

### 2.3. Statistics

In this study, we used SPSS (version 25.0, SPSS Inc., New York, USA) for statistical analysis. A *P*-value of .05 was considered statistically significant. To determine the normality of continuous variables, we used the Shapiro–Wilk test. If the data followed a normal distribution, we expressed them as mean ± standard deviation (SD) and used a t-test for statistical analysis. However, if the data did not follow a normal distribution, we used the median and interquartile range (IQR), and the Mann–Whitney *U* test was used to perform statistical analysis between groups. For count data, expressed as a number and percentage, we used the Chi-square and Fisher’s exact tests to compare the between-group differences.

To find the best prognostic indicator for patients with ACS, we used binary logistic regression analysis. We also used receiver operating characteristic (ROC) analysis to determine the optimal cutoff values for continuous variables, such as PLR. The Youden index (sensitivity + specificity - 1) was used to determine the maximum value, and the cutoff value was classified as low versus high risk. The area under the ROC curve (area under the curve) was used to determine the diagnostic ability, which ranged from 0 to 100%, with a higher area indicating better ability. These statistical analyses allowed us to identify the independent risk factors for blister formation in ACS patients and develop individualized prevention and management strategies.

## 3. Result

In our research, we analyzed a cohort of 206 individuals, comprising 180 males and 26 females, to investigate blister formation in ACS patients. The incidence of blister formation was observed at 21.8%, with 45 patients exhibiting blisters and 161 without.

Detailed analysis, as presented in Table 1, revealed no significant variances in age, gender, Body Mass Index, mechanism of injury, latency from injury to hospital admission, incidence of multiple fractures, smoking habits, ASA classification, and the application of dehydrating agents between the BG and the CG, all yielding *P*-values >.05. Conversely, noteworthy distinctions were detected in relation to seasonal factors (*P* = .007), referral status (*P* = .024), and a history of alcohol use (*P* = .020) between the groups. Specifically, the data highlighted an increased propensity for blister development during the colder months of winter and spring. Additionally, a greater percentage of patients in the BG were referred from other hospitals in comparison to the CG. Intriguingly, individuals in the CG demonstrated a higher likelihood of alcohol consumption, suggesting that while seasonal variations and referral processes might escalate the risk of blisters in ACS scenarios, a history of alcohol consumption could potentially exert a protective influence against their formation.

**Table 1:** Demographics data of patients with and without blisters.

Characteristics	Blister group (n = 45)	Non-blister group (n = 161)	*p*
Age, yrs	39.0 (30.0–54.8)	40.0 (28.5–51.0)	.742
Gender, n (%)			.503
Male	38 (84.4%)	142 (88.2%)	
Female	7 (15.6%)	19 (11.8%)	
Body mass index, kg/m^2^	24.5 (22.7–27.3)	24.5 (22.5–26.7)	.774
<24	16 (35.6%)	55 (34.2%)	.975
24–28	22 (48.9%)	79 (49.1%)	
>28	7 (15.6%)	27 (16.8%)	
Mechanism of injury, n (%)			.213
Car crash injury	9 (20.0%)	35 (21.7%)	
Fall injury	12 (26.7%)	20 (12.4%)	
Crush injury	9 (20.0%)	46 (28.6%)	
Hurt by a heavy object	5 (11.1%)	18 (11.2%)	
Unknown trauma	10 (22.2%)	42 (26.1%)	
Seasonal factors			.007*
Winter and spring	25 (55.6%)	54 (33.5%)	
Summer and fall	20 (44.4%)	107 (66.5%)	
Referral			.024*
Yes	40 (88.9%)	117 (72.7%)	
No	5 (11.1%)	44 (27.3%)	
Time from injury to admission	6 (3.3–14.3)	6 (4–11)	.859
Multiple fracture, n (%)			.681
Yes	27 (60.0%)	102 (63.4%)	
No	18 (40.0%)	59 (36.6%)	
Smoking history, n (%)			.270
Yes	5 (11.1%)	29 (18.0%)	
No	40 (88.9%)	132 (82.0%)	
Alcohol history, n (%)			.020*
Yes	2 (4.4%)	24 (14.9%)	
No	43 (95.6%)	137 (85.1%)	
Dehydrating agent or not, n (%)			.272
Yes	104 (64.6%)	33 (73.3%)	
No	57 (35.4%)	12 (26.7%)	
ASA			.320
1–2	38 (90.5%)	130 (84.4%)	
3–4	4 (99.5%)	24 (15.6%)	

ASA = American Society of Anesthesiologists; Values are presented as the number (%) or the median (interquartile range).

* *P* < .05, statistical significance.

Table 2 offers an in-depth comparison of comorbidity profiles between the 2 study groups, revealing no statistically significant disparities in the prevalence of conditions such as arrhythmia, coronary heart disease, diabetes, hypertension, cerebral infarction, hypoproteinemia, and anemia, with all p-values exceeding 0.05. Conversely, Table 3 delineates the laboratory findings, where notable distinctions emerge. Specifically, the MLR and PLR exhibited significantly elevated levels in the BG compared to the CG, with *P*-values of .011 and .048, respectively. Similarly, the TC levels were significantly higher in the BG (*P* = .027). In contrast, the analysis of other laboratory parameters revealed no significant differences between the groups, underscoring the particular relevance of MLR, PLR, and TC levels as distinguishing factors.

**Table 2:** Comorbidities data of patients with and without blisters.

Comorbidities	Blister group (n = 45)	Non-blister group (n = 161)	*P*
Arrhythmia, n (%)			.516
Yes	0 (0.0%)	5 (3.1%)	
No	45 (100.0%)	156 (96.9%)	
Coronary heart disease, n (%)			.708
Yes	2 (4.4%)	12 (7.5%)	
No	43 (95.6%)	149 (92.5%)	
Hypertension, n (%)			.781
Yes	6 (13.3%)	19 (11.8%)	
No	39 (86.7%)	142 (88.2%)	
Diabetes, n (%)			.338
Yes	0 (0.0%)	7 (4.3%)	
No	45 (100.0%)	154 (95.7%)	
**Cerebral infarction, n (%**)			1.000
Yes	2 (4.4%)	6 (3.7%)	
No	43 (95.6%)	155 (96.3%)	
**Hypoproteinemia, n (%**)			.871
Yes	6 (13.3%)	23 (14.3%)	
No	39 (86.7%)	138 (85.7%)	
**Anemia**			.780
Yes	13 (28.9%)	50 (31.1%)	
No	32 (71.1%)	111 (68.9%)	

Values are presented as the number(%) or the median(interquartile range).

*P < .05, statistical significance.

**Table 3:** Laboratory results of patients with and without blisters.

Laboratory results	Blister group (n = 45)	Non-blister group (n = 161)	*P*
BAS	0.04 (0.01–0.08)	0.04 (0.01–0.07)	.730
EOS	0.09 (0.02–0.16)	0.11 (0.05–0.17)	.449
HCT	38.23 (33.95–43.13)	38.82 (34.58–43.47)	.869
HGB	130.10 (114.40–145.15)	131.20 (117.35–148.00)	.888
IMM	0.18 (0.04–0.20)	0.15 (0.05–0.19)	.988
LYM	1.37 (1.06–1.66)	1.54 (1.06–1.90)	.189
MCH	31.33 (30.25–32.49)	31.27 (30.08–32.21)	.368
MCHC	337.43 ± 10.79	337.82 ± 9.94	.816
MCV	92.77 (90.12–95.71)	92.34 (89.15–95.40)	.337
MON	0.97 (0.62–1.17)	0.81 (0.57–1.07)	.055
MPV	9.00 (8.09–9.94)	8.97 (7.88–9.81)	.651
NEU	10.96 (7.92–15.76)	10.88 (6.89–13.22)	.481
PLT	219.36 ± 51.87	208.62 ± 72.49	.267
RBC	4.28 (3.70–4.72)	4.33 (3.71–4.84)	.870
WBC	12.87 (10.16–18.14)	13.15 (9.55–16.50)	.555
NLR	59.23 (45.29–81.99)	54.83 (37.62–77.61)	.197
MLR	0.66 (0.53–1.04)	0.54 (0.38–0.84)	.011*
PLR	164.97 (122.88–206.82)	137.32 (104.01–189.85)	.048*
ALB	36.44 (33.53–40.60)	36.29 (35.55–42.35)	.940
ALP	67.69 (56.00–70.00)	69.18 (56.00–70.10)	.165
ALT	39.00 (21.50–48.21)	37.00 (24.00–50.02)	.644
AST	63.00 (28.00–100.27)	48.00 (27.00–112.49)	.913
Ca	2.08 (2.08–2.22)	2.09 (2.09–2.19)	.089
CHE	6.61 (6.61–7.41)	6.63 (5.89–7.02)	.518
CK	4965.41 (998.95–4965.41)	2993.25 (381.75–5347.68)	.768
CL	104.48 (101.30–104.84)	104.26 (102.70–105.95)	.466
CREA	68.16 (60.87–73.15)	68.09 (56.96–69.55)	.098
DBIL	5.51 (3.47–6.89)	5.45 (3.50–5.98)	.083
GLOB	22.22 (20.95–23.90)	22.23 (20.30–23.40)	.318
GLU	8.90 (6.40–10.39)	9.00 (6.13–10.16)	.362
K	3.92 (3.75–4.23)	3.92 (3.67–4.09)	.139
LDH	615.31 (257.91–615.31)	628.00 (260.93–689.60)	.269
Mg	0.83 (0.77–0.83)	0.82 (0.77–0.86)	.373
Na	137.99 (135.79–138.80)	137.79 (136.65–139.60)	.638
OSM	270.83 (267.55–270.97)	270.29 (267.45–271.55)	.184
P	1.11 (0.97–1.18)	1.11 (1.01–1.19)	.082
TC	3.46 (2.83–4.08)	3.42 (3.05–3.65)	.027*
TCO2	23.70 (21.71–25.00)	23.70 (22.52–25.80)	.743
TG	1.32 (0.97–1.32)	1.31 (0.85–1.31)	.165
TP	58.44 (57.60–67.05)	58.57 (54.15–65.16)	.670
UA	313.00 (256.50–314.21)	318.99 (248.50–363.00)	.088
UREA	5.53 (4.49–5.90)	5.48 (4.30–5.76)	.105
APTT	29.38 (26.55–30.35)	29.56 (26.70–30.90)	.696
FIB	2.77 (2.38–3.10)	2.77 (2.23–3.01)	.733
INR	1.09 (1.02–1.13)	1.10 (1.02–1.14)	.457
PT	12.40 (11.80–13.00)	12.57 (11.40–13.00)	.488
TT	16.00 (14.10–16.73)	16.40 (14.45–17.25)	.915

Values are presented as the number (%) or the median (interquartile range).

ALB = albumin, ALP = alkaline phosphatase, ALT = alanine transaminase, APTT = activated partial thromboplastin time, AST = aspartate aminotransferase, BAS = basophil, Ca = calcium, CHE = cholinesterase, CK = creatine kinase, CREA = creatinine, DBIL = direct bilirubin, EOS = eosinophil, FIB = Fibrinogen, GLOB = globulin, GLU = glucose, HCT = hematocrit, HGB = hemoglobin, IMM = immature, INR = international normalized ratio, LDH = lactic dehydrogenase, LYM = lymphocyte, MCH = mean corpusular hemoglobin, MCHC = mean corpusular hemoglobin concentration, MCV = mean corpuscular volume, MON = monocyte, MPV = mean platelet volume, NEU = neutrophil, OSM = osmotic pressure, PLT = platelet, PT = prothrombin time, RBC = red blood cell, TC = total cholesterol, TCO2 = total carbon dioxide, TG = triglyceride, TT = thrombin time, UA = uric acid, UREA = ureophil, WBC = White blood cell

* *P* < .05, statistical significance.

Logistic regression analysis shed light on multiple factors significantly associated with an elevated risk of blister development in patients suffering from ACS. Notably, the incidence of ACS during the colder seasons of winter or spring emerged as a significant risk factor (*P* = .007, OR = 2.690, 95% CI [1.308–5.534]). Additionally, a history of being referred from other healthcare facilities significantly increased the risk (*P* = .009, OR = 4.235, 95% CI [1.432–12.527]), as did elevated PLR levels (*P* = .036, OR = 1.005, 95% CI [1.000–1.009]). Conversely, our findings highlighted a history of alcohol consumption as a protective factor against the development of blisters in ACS cases (*P* = .039, OR = 0.207, 95% CI [0.046–0.927]), as detailed in Table 4.

**Table 4:** Binary logistic regression analysis of variables associated with blisters.

Characteristics	OR	95% CI	*P*
Season	2.690	1.308–5.534	.007*
Referral	4.235	1.432–12.527	.009*
Alcohol	0.207	0.046–0.927	.039*
PLR	1.005	1.000–1.009	.036*

Values are presented as the number (%) or the median (interquartile range).

* *P* < .05, statistical significance.

**Figure 2.**
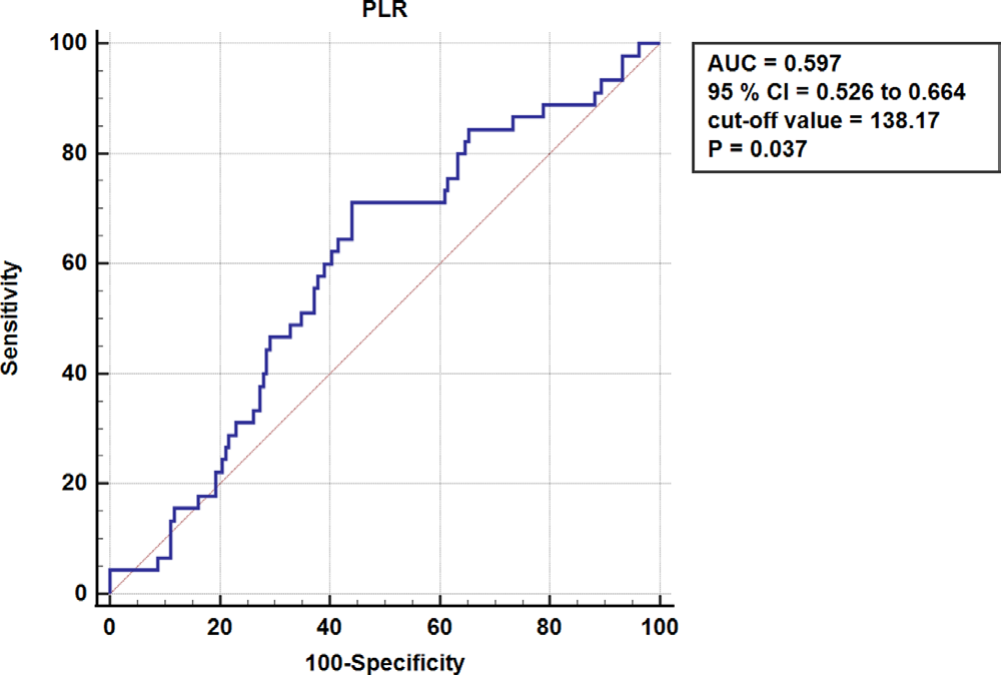
Receiver operating characteristic curve analysis of PLR.

Figure 2 illustrates the determinants pinpointed through ROC curve analysis. Intriguingly, a PLR threshold of 138 was delineated as the predictive cutoff value for blister formation among ACS patients (*P* = .037, area under the curve = 0.597, 95% CI [0.526–0.664]), underlining the diagnostic utility of PLR in anticipating blister occurrence within this patient cohort.

## 4. Discussion

ACS manifests as a severe complication stemming from fractures or other traumas confined within a closed fascial compartment.^[1,2]^ At its core, the pathogenesis of ACS involves reperfusion-induced edema, precipitating an escalation in compartment pressure and consequent tissue necrosis. Without timely and accurate diagnosis, this cascade can lead to catastrophic outcomes, including irreversible damage to nerves and muscles, necessitation of limb amputation, or in dire circumstances, fatality.^[5,6]^ A notable manifestation associated with the augmented pressure within these compartments is the formation of blisters, predominantly observed in the legs and forearms.^[3,4]^ Hospitalized patients exhibit a 2.9% incidence rate of blister occurrence, particularly following high-energy orthopedic traumas in regions where the skin is in close adherence to the underlying bone with scant subcutaneous fat padding.^[12]^ The presence of blisters poses significant clinical challenges, notably the postponement of surgical interventions and heightened susceptibility to infections.^[13]^

The prevalent incidence and the adverse prognosis associated with blister formation underscore the imperative need for the identification of predictive factors and the implementation of prophylactic strategies. Despite ongoing inquiries into the pathophysiology of blister development,^[14]^ there remains a notable paucity of research specifically exploring the predictors of blister occurrence in individuals afflicted with ACS. This study pioneers the exploration of risk factors contributing to blister formation in the context of ACS, aiming to bridge this critical knowledge gap and enhance patient care protocols.

In this investigation, we noted a blister occurrence rate of 21.8%, with 45 out of 206 patients affected. Through univariate analysis, we pinpointed a series of predictors correlated with the emergence of blisters, including seasonal variations, patient referral history, alcohol consumption habits, elevated TC levels, and increased MLR and PLR. Further, logistic regression analysis underscored the significance of admissions during the colder months of winter or spring, a history of patient referrals, and elevated PLR values as critical predictors for blister development. Moreover, ROC curve analysis meticulously determined that a PLR threshold of 138 serves as a predictive marker for blister occurrence.

In our analysis, “referral” is delineated as the process through which patients are shuttled among various medical establishments for additional assessments and treatment endeavors before being admitted to our institution. Notably, our findings revealed a pronounced susceptibility to blister development among referred patients, who demonstrated a 4.2-fold increased risk compared to their directly admitted counterparts. The referral pathway not only extends the pre-hospitalization duration but also signifies that patients have been subject to numerous relocations, iterative imaging, and physical evaluations. A subset of participants in our research experienced multiple referrals across different healthcare facilities for identical tests prior to their eventual admission to our hospital. Such scenarios invariably involve continuous patient transfers, repeated manipulation of the injured area, extended wait times, and recurrent assessments in varying clinical settings, all of which could postpone the initiation of early therapeutic measures like the application of dehydrants or ice to mitigate soft tissue swelling, thereby intensifying soft tissue trauma and edema. Consistent with prior studies,^[16,17]^ our investigation corroborates that the trauma inflicted by ACS can precipitate soft tissue edema and inflammation, potentially culminating in dermal hypoxia, further soft tissue damage, and in certain instances, cutaneous and muscular necrosis. Endorsing this viewpoint, Nelson et al have advocated for prompt fracture reduction and stabilization to diminish the occurrence of fracture blisters.^[18]^ Similarly, Frank et al have emphasized the criticality of immediate limb immobilization post-injury, preferably at the accident scene, as an initial therapeutic step to avert additional soft tissue harm.^[19]^ These expert recommendations resonate with our observations, underscoring the intricate link between blister formation in ACS patients and the frequency of referrals. Hence, enhancing the diagnostic and treatment capabilities of primary care facilities, implementing early temporary external fixation, and ensuring swift data transfer among referral networks emerge as vital strategies to mitigate blister incidence in the ACS demographic.

We discerned a marked distinction in the PLR between the Blister Group (BG) and the Control Group (CG), with elevated PLR levels observed within the BG. Despite the absence of prior studies directly linking PLR with blister occurrence, insights into the relationship between trauma severity and blister formation provide a compelling context for our findings. Maria et al highlight the direct correlation between the magnitude of energy dissipated at the injury site and the subsequent severity of soft tissue damage, a key factor exacerbating Acute Compartment Syndrome (ACS).^[20]^ Furthermore, it is posited that the extent of soft tissue damage following fractures bears a direct relationship to the development of blisters,^[21]^ situating blisters in ACS patients within the broader category of fracture-induced blisters, indicative of trauma severity.

The PLR has emerged as a notable marker for systemic inflammatory response across various clinical scenarios, including fractures, oncological conditions, and instances of polytrauma.^[22–25]^ Research indicates that significant trauma triggers heightened platelet activation, thereby initiating a cascade of coagulation and immune responses.^[26]^ Wang et al corroborate this, demonstrating the postoperative PLR as a robust biomarker closely associated with the trauma severity in patients undergoing treatment for bicondylar tibial plateau fractures (TPFs).^[22]^ These insights underscore the PLR not merely as a marker of injury severity but as a critical link to blister development, affirming its role in predicting blister occurrence in ACS contexts. Our study reinforces this association, establishing PLR as a predictive factor for blister development among ACS patients. This prognostic capability enables clinicians to anticipate the likelihood of blister formation more accurately and to tailor preventive measures accordingly.

Additionally, we explored the MLR as an indicator of inflammation in the context of blister formation. Although univariate analysis identified a correlation between MLR and blister occurrence, logistic regression analysis did not substantiate MLR as an independent risk factor, further accentuating the unique prognostic value of PLR in this clinical setting.

Our study found that seasonal factors, particularly in winter and spring, were an independent risk factor for blister formation in patients with ACS. Shijiazhuang and Baoding, where our study was conducted, are located in the north of China, where winter and spring temperatures can range from –3.0°C to 7.0°C. Seasonal changes in temperature can potentially influence fracture-related disorders. Previous studies have shown a link between fractures and temperature, with Cecilie et al suggesting that cold ambient temperatures can increase the incidence of forearm and hip fractures, as well as post-hip fracture mortality.^[27]^ Additionally, Kinga et al found that changes in fracture incidence were related to season (warmer vs colder) and the increase in mean temperature during the observation period.^[28]^ This could be due to the need for thicker clothing in cold weather, resulting in repeated movements due to the inconvenience of exposing the affected limb during the emergency examination. Furthermore, cold weather may negatively impact patients’ exposure to the external environment at the time of injury and during ambulance transport, potentially leading to hypothermia, which can have detrimental effects on trauma patients’ metabolic and coagulation systems. Simple interventions, such as passive and active external warming, as well as core warming with warmed IV fluids, can help maintain thermostasis and prevent hypothermia.^[29]^ However, studies have reported that exposure to the cold is common in prehospital care, and patients often experience discomfort from exposure to the cold.^[30]^ Therefore, maintaining a comfortable temperature for the patient during transport is critical. Our findings suggest that evaluating whether a patient has been exposed to cold can aid in predicting the occurrence of blisters in ACS patients, which can inform perioperative care and surgical planning, preventing unnecessary surgery and perioperative complications related to blister formation.

We also identified an indicator that acted as a protective factor against blister formation in ACS patients. According to logistic regression analysis, patients with a history of alcohol consumption were less likely to develop blisters. Previous research has established a correlation between alcohol consumption and bone mineral density,^[31–33]^ with patients who have a history of alcohol consumption tending to have higher bone mineral density. Lower bone density has been associated with the development of comminuted fractures, which can cause significant soft tissue injury. As mentioned earlier, severe soft tissue injury is positively correlated with the development of blisters, which may explain the protective role of a history of alcohol consumption in delaying blister formation.

Despite the significant findings of this study, several limitations should be noted. First, the study’s retrospective design limited our ability to capture all potential factors that may be associated with blister formation, such as surgical history. Second, the sample size of ACS patients was relatively small, and we did not perform subgroup analysis based on blister location, such as lower limb or upper limb blisters. Third, as with any multivariate study, we could not account for all potential confounding variables, and residual confounding remains a concern. Future studies with larger sample sizes, prospective designs, and more comprehensive data collection are needed to confirm and expand upon our findings. Besides, we acknowledge that the retrospective nature of our study precluded the application of blinding during data collection, which could introduce biases in interpreting patient outcomes or exposure status. Future prospective studies are encouraged to incorporate blinding methods to enhance reliability and minimize biases. Furthermore, due to the retrospective design and reliance on electronic medical records, specific data such as alcohol intake and precise blood sugar levels may not be consistently available or recorded. The absence of such data could potentially affect the interpretation of blister formation among ACS patients.

To conclude, our study identified several factors associated with blister formation in patients with ACS, including seasonal factors, referral, MLR, PLR, and TC. Logistic regression analysis revealed that patients who developed ACS in the winter or spring, those with a history of referral, and those with higher PLR levels were independent predictors of blister formation. Furthermore, we determined that 138 was the cutoff value for PLR to predict blister formation. Interestingly, a history of alcohol consumption was found to be a protective factor against blister formation. These findings provide valuable insights into the risk factors for blister development in ACS patients and offer a personalized risk assessment, allowing for timely and targeted interventions to prevent blister formation and reduce the incidence of complications.

## Acknowledgments

We appreciate the great help from the 3rd Hospital of Hebei Medical University and Baoding No.1 Central Hospital.

## Author contributions

**Writing – original draft:** Shuo Yang.

**Writing – review & editing:** Yubin Long, Junfei Guo, Zhiyong Hou.
